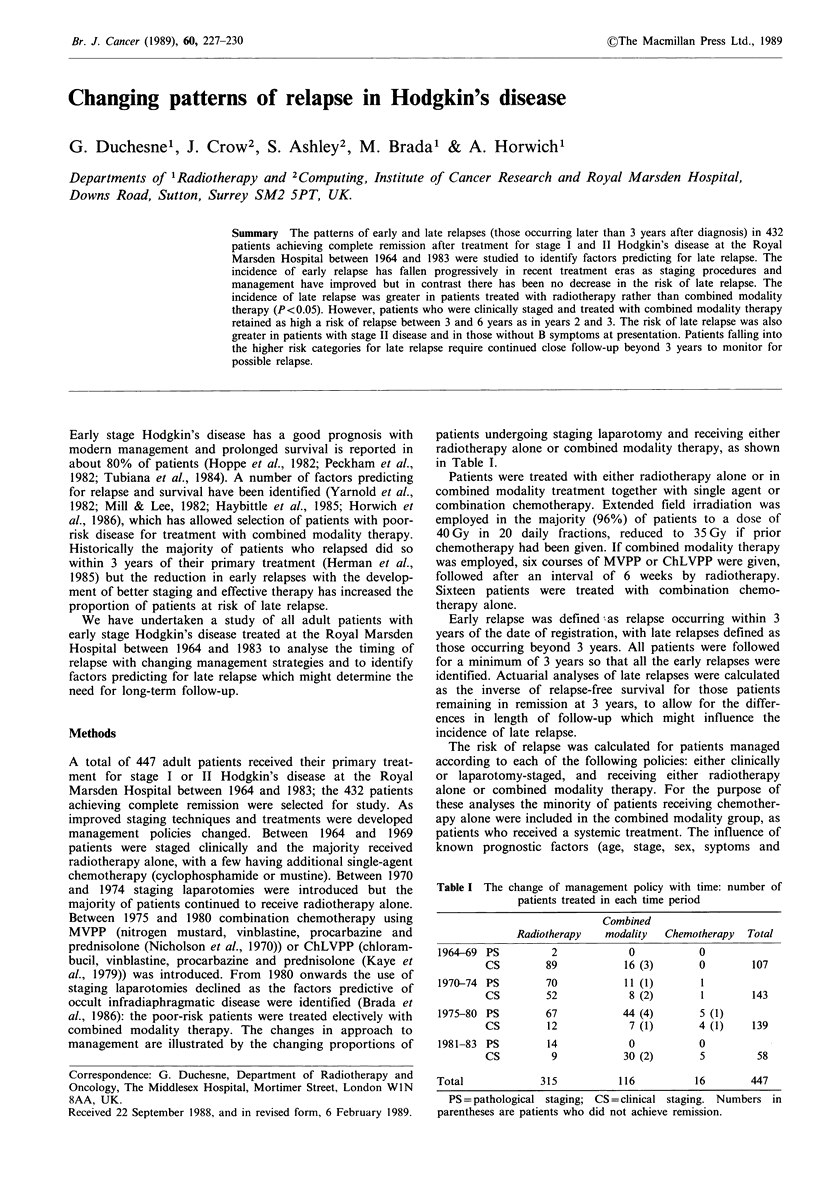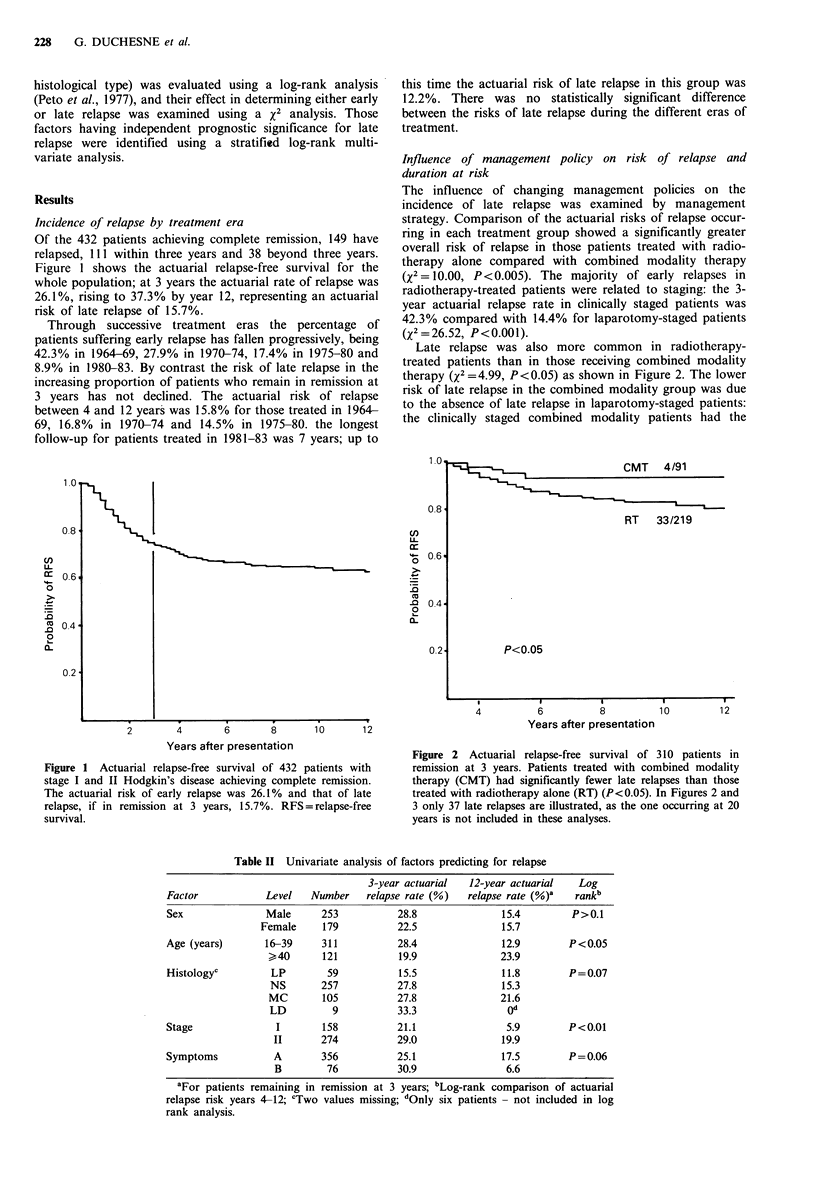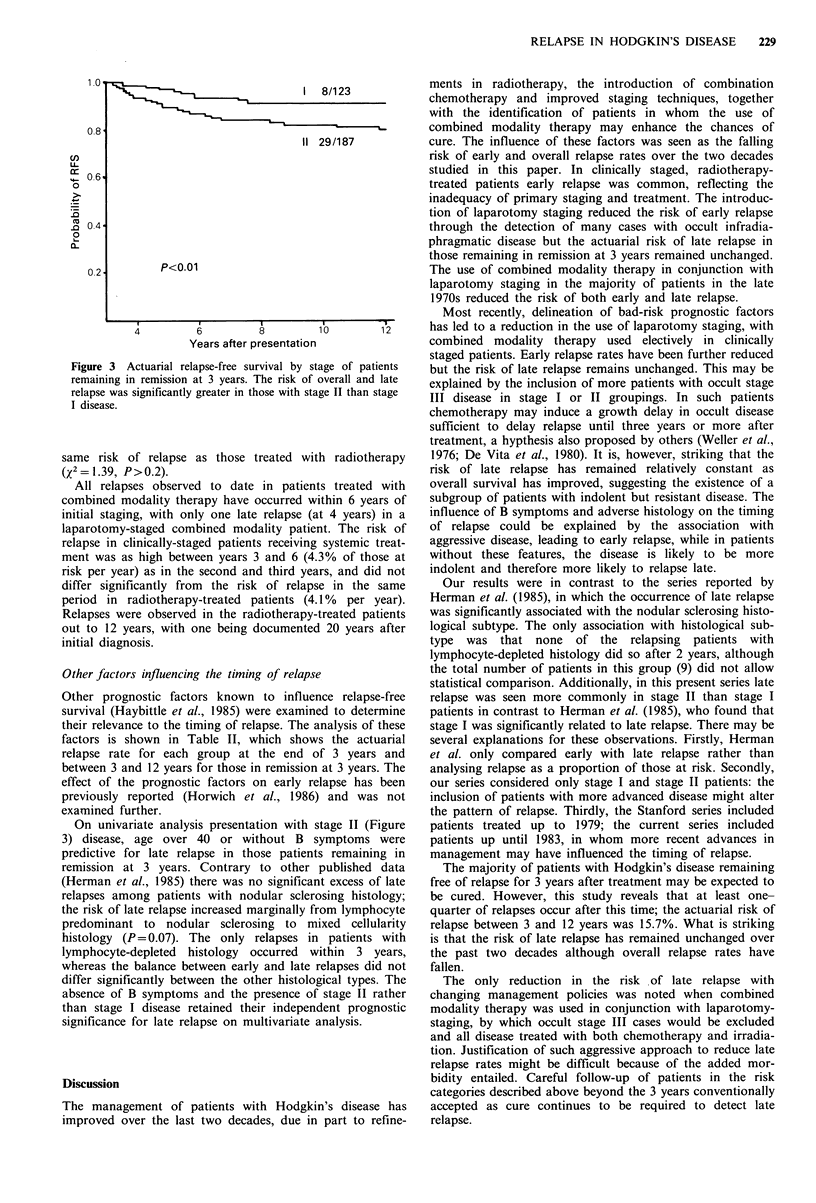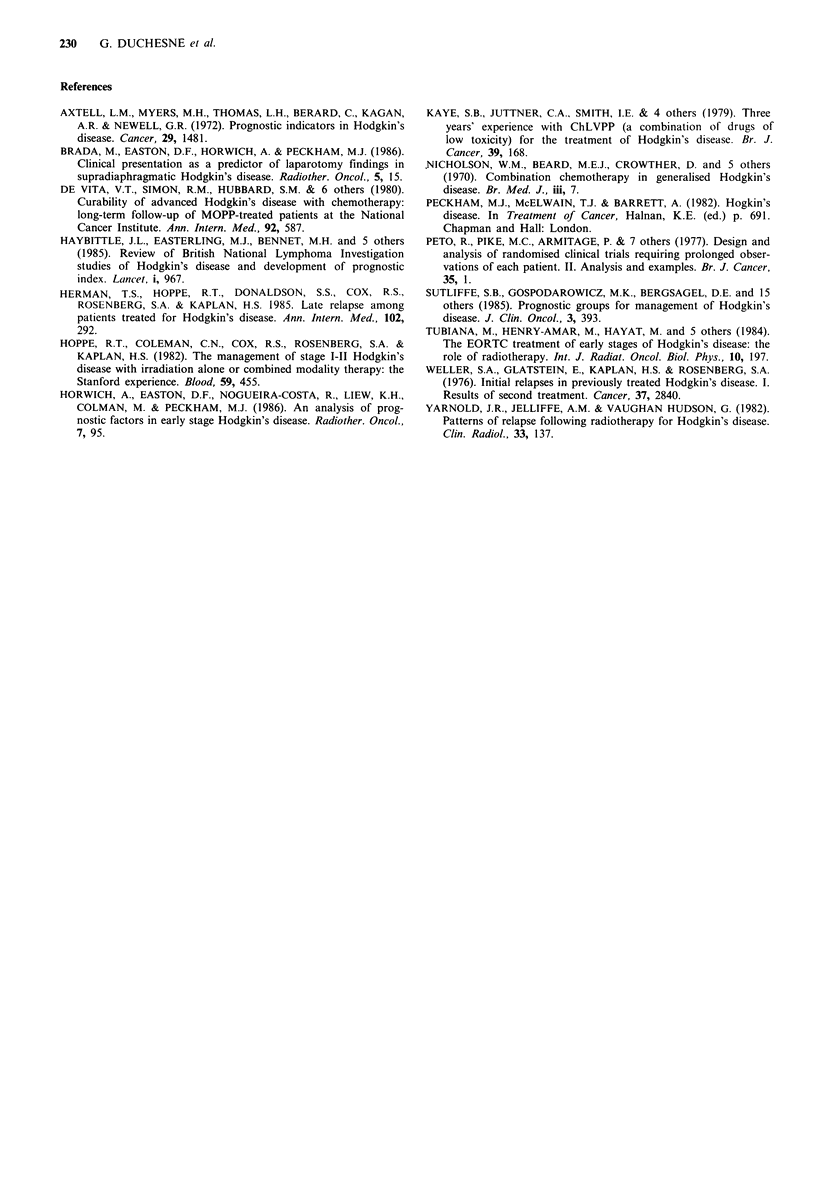# Changing patterns of relapse in Hodgkin's disease.

**DOI:** 10.1038/bjc.1989.257

**Published:** 1989-08

**Authors:** G. Duchesne, J. Crow, S. Ashley, M. Brada, A. Horwich

**Affiliations:** Department of Radiotherapy, Royal Marsden Hospital, Surrey, UK.

## Abstract

The patterns of early and late relapses (those occurring later than 3 years after diagnosis) in 432 patients achieving complete remission after treatment for stage I and II Hodgkin's disease at the Royal Marsden Hospital between 1964 and 1983 were studied to identify factors predicting for late relapse. The incidence of early relapse has fallen progressively in recent treatment eras as staging procedures and management have improved but in contrast there has been no decrease in the risk of late relapse. The incidence of late relapse was greater in patients treated with radiotherapy rather than combined modality therapy (P less than 0.05). However, patients who were clinically staged and treated with combined modality therapy retained as high a risk of relapse between 3 and 6 years as in years 2 and 3. The risk of late relapse was also greater in patients with stage II disease and in those without B symptoms at presentation. Patients falling into the higher risk categories for late relapse require continued close follow-up beyond 3 years to monitor for possible relapse.


Br. J. Cancer (1989), 60, 227–230 ©The Macmillan Press Ltd., 1989

### Changing patterns of relapse in Hodgkin's disease

G. Duchesne[1], J. Crow[2], S. Ashley[2], M. Brada[1] & A. Horwich[1]

*Departments of [1]Radiotherapy and [2]Computing, Institute of Cancer Research and Royal Marsden Hospital, Downs Road, Sutton, Surrey SM2 5PT, UK.*

**Summary** The patterns of early and late relapses (those occurring later than 3 years after diagnosis) in 432 patients achieving complete remission after treatment for stage I and II Hodgkin's disease at the Royal Marsden Hospital between 1964 and 1983 were studied to identify factors predicting for late relapse. The incidence of early relapse has fallen progressively in recent treatment eras as staging procedures and management have improved but in contrast there has been no decrease in the risk of late relapse. The incidence of late relapse was greater in patients treated with radiotherapy rather than combined modality therapy ($P<0.05$). However, patients who were clinically staged and treated with combined modality therapy retained as high a risk of relapse between 3 and 6 years as in years 2 and 3. The risk of late relapse was also greater in patients with stage II disease and in those without B symptoms at presentation. Patients falling into the higher risk categories for late relapse require continued close follow-up beyond 3 years to monitor for possible relapse.

Early stage Hodgkin's disease has a good prognosis with modern management and prolonged survival is reported in about 80% of patients (Hoppe *et al.*, 1982; Peckham *et al.*, 1982; Tubiana *et al.*, 1984). A number of factors predicting for relapse and survival have been identified (Yarnold *et al.*, 1982; Mill & Lee, 1982; Haybittle *et al.*, 1985; Horwich *et al.*, 1986), which has allowed selection of patients with poor-risk disease for treatment with combined modality therapy. Historically the majority of patients who relapsed did so within 3 years of their primary treatment (Herman *et al.*, 1985) but the reduction in early relapses with the development of better staging and effective therapy has increased the proportion of patients at risk of late relapse.

We have undertaken a study of all adult patients with early stage Hodgkin's disease treated at the Royal Marsden Hospital between 1964 and 1983 to analyse the timing of relapse with changing management strategies and to identify factors predicting for late relapse which might determine the need for long-term follow-up.

### Methods

A total of 447 adult patients received their primary treatment for stage I or II Hodgkin's disease at the Royal Marsden Hospital between 1964 and 1983; the 432 patients achieving complete remission were selected for study. As improved staging techniques and treatments were developed management policies changed. Between 1964 and 1969 patients were staged clinically and the majority received radiotherapy alone, with a few having additional single-agent chemotherapy (cyclophosphamide or mustine). Between 1970 and 1974 staging laparotomies were introduced but the majority of patients continued to receive radiotherapy alone. Between 1975 and 1980 combination chemotherapy using MVPP (nitrogen mustard, vinblastine, procarbazine and prednisolone (Nicholson *et al.*, 1970)) or ChLVPP (chlorambucil, vinblastine, procarbazine and prednisolone (Kaye *et al.*, 1979)) was introduced. From 1980 onwards the use of staging laparotomies declined as the factors predictive of occult infradiaphragmatic disease were identified (Brada *et al.*, 1986): the poor-risk patients were treated electively with combined modality therapy. The changes in approach to management are illustrated by the changing proportions of patients undergoing staging laparotomy and receiving either radiotherapy alone or combined modality therapy, as shown in Table I.

Patients were treated with either radiotherapy alone or in combined modality treatment together with single agent or combination chemotherapy. Extended field irradiation was employed in the majority (96%) of patients to a dose of 40 Gy in 20 daily fractions, reduced to 35 Gy if prior chemotherapy had been given. If combined modality therapy was employed, six courses of MVPP or ChLVPP were given, followed after an interval of 6 weeks by radiotherapy. Sixteen patients were treated with combination chemotherapy alone.

Early relapse was defined as relapse occurring within 3 years of the date of registration, with late relapses defined as those occurring beyond 3 years. All patients were followed for a minimum of 3 years so that all the early relapses were identified. Actuarial analyses of late relapses were calculated as the inverse of relapse-free survival for those patients remaining in remission at 3 years, to allow for the differences in length of follow-up which might influence the incidence of late relapse.

The risk of relapse was calculated for patients managed according to each of the following policies: either clinically or laparotomy-staged, and receiving either radiotherapy alone or combined modality therapy. For the purpose of these analyses the minority of patients receiving chemotherapy alone were included in the combined modality group, as patients who received a systemic treatment. The influence of known prognostic factors (age, stage, sex, syptoms and

**Table I** The change of management policy with time: number of patients treated in each time period

| | | *Radiotherapy* | *Combined modality* | *Chemotherapy* | *Total* |
|---|---|---|---|---|---|
| 1964–69 | PS | 2 | 0 | 0 | |
| | CS | 89 | 16 (3) | 0 | 107 |
| 1970–74 | PS | 70 | 11 (1) | 1 | |
| | CS | 52 | 8 (2) | 1 | 143 |
| 1975–80 | PS | 67 | 44 (4) | 5 (1) | |
| | CS | 12 | 7 (1) | 4 (1) | 139 |
| 1981–83 | PS | 14 | 0 | 0 | |
| | CS | 9 | 30 (2) | 5 | 58 |
| Total | | 315 | 116 | 16 | 447 |

PS = pathological staging; CS = clinical staging. Numbers in parentheses are patients who did not achieve remission.

Correspondence: G. Duchesne, Department of Radiotherapy and Oncology, The Middlesex Hospital, Mortimer Street, London W1N 8AA, UK.
Received 22 September 1988, and in revised form, 6 February 1989.

histological type) was evaluated using a log-rank analysis (Peto et al., 1977), and their effect in determining either early or late relapse was examined using a $\chi^2$ analysis. Those factors having independent prognostic significance for late relapse were identified using a stratified log-rank multivariate analysis.

### Results

### Incidence of relapse by treatment era

Of the 432 patients achieving complete remission, 149 have relapsed, 111 within three years and 38 beyond three years. Figure 1 shows the actuarial relapse-free survival for the whole population; at 3 years the actuarial rate of relapse was 26.1%, rising to 37.3% by year 12, representing an actuarial risk of late relapse of 15.7%.

Through successive treatment eras the percentage of patients suffering early relapse has fallen progressively, being 42.3% in 1964–69, 27.9% in 1970–74, 17.4% in 1975–80 and 8.9% in 1980–83. By contrast the risk of late relapse in the increasing proportion of patients who remain in remission at 3 years has not declined. The actuarial risk of relapse between 4 and 12 years was 15.8% for those treated in 1964–69, 16.8% in 1970–74 and 14.5% in 1975–80. the longest follow-up for patients treated in 1981–83 was 7 years; up to this time the actuarial risk of late relapse in this group was 12.2%. There was no statistically significant difference between the risks of late relapse during the different eras of treatment.

### Influence of management policy on risk of relapse and duration at risk

The influence of changing management policies on the incidence of late relapse was examined by management strategy. Comparison of the actuarial risks of relapse occurring in each treatment group showed a significantly greater overall risk of relapse in those patients treated with radiotherapy alone compared with combined modality therapy ($\chi^2=10.00$, $P<0.005$). The majority of early relapses in radiotherapy-treated patients were related to staging: the 3-year actuarial relapse rate in clinically staged patients was 42.3% compared with 14.4% for laparotomy-staged patients ($\chi^2=26.52$, $P<0.001$).

Late relapse was also more common in radiotherapy-treated patients than in those receiving combined modality therapy ($\chi^2=4.99$, $P<0.05$) as shown in Figure 2. The lower risk of late relapse in the combined modality group was due to the absence of late relapse in laparotomy-staged patients: the clinically staged combined modality patients had the

**Figure 1** Actuarial relapse-free survival of 432 patients with stage I and II Hodgkin's disease achieving complete remission. The actuarial risk of early relapse was 26.1% and that of late relapse, if in remission at 3 years, 15.7%. RFS = relapse-free survival.

**Figure 2** Actuarial relapse-free survival of 310 patients in remission at 3 years. Patients treated with combined modality therapy (CMT) had significantly fewer late relapses than those treated with radiotherapy alone (RT) ($P<0.05$). In Figures 2 and 3 only 37 late relapses are illustrated, as the one occurring at 20 years is not included in these analyses.

**Table II** Univariate analysis of factors predicting for relapse

| Factor | Level | Number | 3-year actuarial relapse rate (%) | 12-year actuarial relapse rate (%)[a] | Log rank[b] |
|---|---|---|---|---|---|
| Sex | Male | 253 | 28.8 | 15.4 | $P>0.1$ |
| | Female | 179 | 22.5 | 15.7 | |
| Age (years) | 16–39 | 311 | 28.4 | 12.9 | $P<0.05$ |
| | ≥40 | 121 | 19.9 | 23.9 | |
| Histology[c] | LP | 59 | 15.5 | 11.8 | $P=0.07$ |
| | NS | 257 | 27.8 | 15.3 | |
| | MC | 105 | 27.8 | 21.6 | |
| | LD | 9 | 33.3 | 0[d] | |
| Stage | I | 158 | 21.1 | 5.9 | $P<0.01$ |
| | II | 274 | 29.0 | 19.9 | |
| Symptoms | A | 356 | 25.1 | 17.5 | $P=0.06$ |
| | B | 76 | 30.9 | 6.6 | |

[a]For patients remaining in remission at 3 years; [b]Log-rank comparison of actuarial relapse risk years 4–12; [c]Two values missing; [d]Only six patients – not included in log rank analysis.

**Figure 3** Actuarial relapse-free survival by stage of patients remaining in remission at 3 years. The risk of overall and late relapse was significantly greater in those with stage II than stage I disease.

same risk of relapse as those treated with radiotherapy ($\chi^2 = 1.39$, $P > 0.2$).

All relapses observed to date in patients treated with combined modality therapy have occurred within 6 years of initial staging, with only one late relapse (at 4 years) in a laparotomy-staged combined modality patient. The risk of relapse in clinically-staged patients receiving systemic treatment was as high between years 3 and 6 (4.3% of those at risk per year) as in the second and third years, and did not differ significantly from the risk of relapse in the same period in radiotherapy-treated patients (4.1% per year). Relapses were observed in the radiotherapy-treated patients out to 12 years, with one being documented 20 years after initial diagnosis.

*Other factors influencing the timing of relapse*

Other prognostic factors known to influence relapse-free survival (Haybittle *et al.*, 1985) were examined to determine their relevance to the timing of relapse. The analysis of these factors is shown in Table II, which shows the actuarial relapse rate for each group at the end of 3 years and between 3 and 12 years for those in remission at 3 years. The effect of the prognostic factors on early relapse has been previously reported (Horwich *et al.*, 1986) and was not examined further.

On univariate analysis presentation with stage II (Figure 3) disease, age over 40 or without B symptoms were predictive for late relapse in those patients remaining in remission at 3 years. Contrary to other published data (Herman *et al.*, 1985) there was no significant excess of late relapses among patients with nodular sclerosing histology; the risk of late relapse increased marginally from lymphocyte predominant to nodular sclerosing to mixed cellularity histology ($P = 0.07$). The only relapses in patients with lymphocyte-depleted histology occurred within 3 years, whereas the balance between early and late relapses did not differ significantly between the other histological types. The absence of B symptoms and the presence of stage II rather than stage I disease retained their independent prognostic significance for late relapse on multivariate analysis.

### Discussion

The management of patients with Hodgkin's disease has improved over the last two decades, due in part to refinements in radiotherapy, the introduction of combination chemotherapy and improved staging techniques, together with the identification of patients in whom the use of combined modality therapy may enhance the chances of cure. The influence of these factors was seen as the falling risk of early and overall relapse rates over the two decades studied in this paper. In clinically staged, radiotherapy-treated patients early relapse was common, reflecting the inadequacy of primary staging and treatment. The introduction of laparotomy staging reduced the risk of early relapse through the detection of many cases with occult infradiaphragmatic disease but the actuarial risk of late relapse in those remaining in remission at 3 years remained unchanged. The use of combined modality therapy in conjunction with laparotomy staging in the majority of patients in the late 1970s reduced the risk of both early and late relapse.

Most recently, delineation of bad-risk prognostic factors has led to a reduction in the use of laparotomy staging, with combined modality therapy used electively in clinically staged patients. Early relapse rates have been further reduced but the risk of late relapse remains unchanged. This may be explained by the inclusion of more patients with occult stage III disease in stage I or II groupings. In such patients chemotherapy may induce a growth delay in occult disease sufficient to delay relapse until three years or more after treatment, a hypthesis also proposed by others (Weller *et al.*, 1976; De Vita *et al.*, 1980). It is, however, striking that the risk of late relapse has remained relatively constant as overall survival has improved, suggesting the existence of a subgroup of patients with indolent but resistant disease. The influence of B symptoms and adverse histology on the timing of relapse could be explained by the association with aggressive disease, leading to early relapse, while in patients without these features, the disease is likely to be more indolent and therefore more likely to relapse late.

Our results were in contrast to the series reported by Herman *et al.* (1985), in which the occurrence of late relapse was significantly associated with the nodular sclerosing histological subtype. The only association with histological subtype was that none of the relapsing patients with lymphocyte-depleted histology did so after 2 years, although the total number of patients in this group (9) did not allow statistical comparison. Additionally, in this present series late relapse was seen more commonly in stage II than stage I patients in contrast to Herman *et al.* (1985), who found that stage I was significantly related to late relapse. There may be several explanations for these observations. Firstly, Herman *et al.* only compared early with late relapse rather than analysing relapse as a proportion of those at risk. Secondly, our series considered only stage I and stage II patients: the inclusion of patients with more advanced disease might alter the pattern of relapse. Thirdly, the Stanford series included patients treated up to 1979; the current series included patients up until 1983, in whom more recent advances in management may have influenced the timing of relapse.

The majority of patients with Hodgkin's disease remaining free of relapse for 3 years after treatment may be expected to be cured. However, this study reveals that at least one–quarter of relapses occur after this time; the actuarial risk of relapse between 3 and 12 years was 15.7%. What is striking is that the risk of late relapse has remained unchanged over the past two decades although overall relapse rates have fallen.

The only reduction in the risk of late relapse with changing management policies was noted when combined modality therapy was used in conjunction with laparotomy-staging, by which occult stage III cases would be excluded and all disease treated with both chemotherapy and irradiation. Justification of such aggressive approach to reduce late relapse rates might be difficult because of the added morbidity entailed. Careful follow-up of patients in the risk categories described above beyond the 3 years conventionally accepted as cure continues to be required to detect late relapse.

### References

AXTELL, L.M., MYERS, M.H., THOMAS, L.H., BERARD, C., KAGAN, A.R. & NEWELL, G.R. (1972). Prognostic indicators in Hodgkin's disease. *Cancer,* **29,** 1481.

BRADA, M., EASTON, D.F., HORWICH, A. & PECKHAM, M.J. (1986). Clinical presentation as a predictor of laparotomy findings in supradiaphragmatic Hodgkin's disease. *Radiother. Oncol.,* **5,** 15.

DE VITA, V.T., SIMON, R.M., HUBBARD, S.M. & 6 others (1980). Curability of advanced Hodgkin's disease with chemotherapy: long-term follow-up of MOPP-treated patients at the National Cancer Institute. *Ann. Intern. Med.,* **92,** 587.

HAYBITTLE, J.L., EASTERLING, M.J., BENNET, M.H. and 5 others (1985). Review of British National Lymphoma Investigation studies of Hodgkin's disease and development of prognostic index. *Lancet,* **i,** 967.

HERMAN, T.S., HOPPE, R.T., DONALDSON, S.S., COX, R.S., ROSENBERG, S.A. & KAPLAN, H.S. 1985. Late relapse among patients treated for Hodgkin's disease. *Ann. Intern. Med.,* **102,** 292.

HOPPE, R.T., COLEMAN, C.N., COX, R.S., ROSENBERG, S.A. & KAPLAN, H.S. (1982). The management of stage I-II Hodgkin's disease with irradiation alone or combined modality therapy: the Stanford experience. *Blood,* **59,** 455.

HORWICH, A., EASTON, D.F., NOGUEIRA-COSTA, R., LIEW, K.H., COLMAN, M. & PECKHAM, M.J. (1986). An analysis of prognostic factors in early stage Hodgkin's disease. *Radiother. Oncol.,* **7,** 95.

KAYE, S.B., JUTTNER, C.A., SMITH, I.E. & 4 others (1979). Three years' experience with ChLVPP (a combination of drugs of low toxicity) for the treatment of Hodgkin's disease. *Br. J. Cancer,* **39,** 168.

NICHOLSON, W.M., BEARD, M.E.J., CROWTHER, D. and 5 others (1970). Combination chemotherapy in generalised Hodgkin's disease. *Br. Med. J.,* **iii,** 7.

PECKHAM, M.J., McELWAIN, T.J. & BARRETT, A. (1982). Hogkin's disease. In *Treatment of Cancer,* Halnan, K.E. (ed.) p. 691. Chapman and Hall: London.

PETO, R., PIKE, M.C., ARMITAGE, P. & 7 others (1977). Design and analysis of randomised clinical trials requiring prolonged observations of each patient. II. Analysis and examples. *Br. J. Cancer,* **35,** 1.

SUTLIFFE, S.B., GOSPODAROWICZ, M.K., BERGSAGEL, D.E. and 15 others (1985). Prognostic groups for management of Hodgkin's disease. *J. Clin. Oncol.,* **3,** 393.

TUBIANA, M., HENRY-AMAR, M., HAYAT, M. and 5 others (1984). The EORTC treatment of early stages of Hodgkin's disease: the role of radiotherapy. *Int. J. Radiat. Oncol. Biol. Phys.,* **10,** 197.

WELLER, S.A., GLATSTEIN, E., KAPLAN, H.S. & ROSENBERG, S.A. (1976). Initial relapses in previously treated Hodgkin's disease. I. Results of second treatment. *Cancer,* **37,** 2840.

YARNOLD, J.R., JELLIFFE, A.M. & VAUGHAN HUDSON, G. (1982). Patterns of relapse following radiotherapy for Hodgkin's disease. *Clin. Radiol.,* **33,** 137.